# Reichsanzeiger-GT: An OCR ground truth dataset based on the historical newspaper “Deutscher Reichsanzeiger und Preußischer Staatsanzeiger” (German Imperial Gazette and Prussian Official Gazette) (1819–1945)

**DOI:** 10.1016/j.dib.2024.110274

**Published:** 2024-03-07

**Authors:** Thomas Schmidt, Jan Kamlah, Stefan Weil

**Affiliations:** University of Mannheim, University Library, Schloss Schneckenhof, 68161 Mannheim

**Keywords:** OCR, Text recognition, Ground truth, Historical newspapers

## Abstract

Reichsanzeiger-GT is a ground truth dataset for OCR training and evaluation based on the historical German newspaper “Deutscher Reichsanzeiger und Preußischer Staatsanzeiger” (German Imperial Gazette and Prussian Official Gazette), which was published from 1819 to 1945 and printed mostly in the typeface Fraktur (Black Letter). The dataset consists of 101 newspaper pages for the years 1820–1939, that cover a wide variety of topics, page layouts (lists, tables, and advertisements) as well as different typefaces. Using the transcription software Transkribus and the open-source OCR engine Tesseract we automatically created and manually corrected layout segmentations and transcriptions for each page, resulting in 65,563 text regions, 412 table regions, 119,429 text lines and 490,679 words. By applying transcription guidelines that preserve the printing conditions, the dataset contains language and printing specific phenomena like the historical use of glyphs like long s (ſ), rotunda r (ꝛ), and historical currency symbols (M, ₰) among others. The dataset is provided in two variants in PAGE XML format. The first one contains ground truth data with table regions transformed to text regions for easier processing. The second variant preserves all table regions. Researchers can reuse this dataset to train new or finetune existing text recognition or layout segmentation models. The dataset can also be used to evaluate the accuracy of existing OCR models. Using specific, community driven transcription guidelines our dataset is easily interoperable and reusable with other datasets based on the same transcription level.

Specifications Table

**Table:** 

Subject	Data Science
Specific subject area	Ground truth data for Optical Character Recognition (OCR) training and evaluation
Data format	Raw, Filtered
Type of data	Text, Image
Data collection	We collected 101 raw images of the corresponding newspaper pages using the digital edition of “Deutscher Reichsanzeiger und Preußischer Staatsanzeiger” [1], shared under the Public Domain Mark 1.0 license. Using the transcription software Transkribus [2], we created an automatic layout segmentation, that was manually corrected afterwards. We then extracted text lines for all pages using the open-source OCR engine Tesseract [3]. The extracted text lines were manually corrected using Transkribus. Each page was corrected twice by different transcribers to assure high data quality.
Data source location	Source of collected images: https://doi.org/10.7801/REICHSANZEIGER
Data accessibility	Repository name: Zenodo Data identification number: https://doi.org/10.5281/zenodo.10144094 Instructions for accessing these data: Images: The Zenodo repository contains a bash script (“download_images.sh”) for downloading all images via CLI. The script can be found in the “data” folder of the repository. Transcriptions in PAGE XML format (with table regions): Transcriptions with annotated table regions can be found in the folder “reichsanzeiger-1820–1939_with-TableRegion” of the Zenodo repository. Transcriptions in PAGE XML format (with table regions transformed to text regions): Transcriptions with annotated table regions transformed into text regions can be found in the folder “reichsanzeiger-1820–1939″ of the Zenodo repository.

## 1 Value of the Data

• The dataset [4] is valuable for training and evaluating text recognition models for historical typefonts (Fraktur and Black Letter). The data captures historical printing conditions, e.g., the use of historical glyphs like long s (ſ), rotunda r (ꝛ), combining Latin small letter E for old German Umlaut (ͤ), and currency symbols (M, ₰). These historical glyphs can be trained with the dataset to improve their recognition in different historical documents.
• The dataset is valuable for training layout segmentation models for historical domains. Especially the segmentation of table regions proves to be a problem for state-of-the-art OCR models due to the heterogeneity of table structures and their varied layouts [5]. Our dataset provides 412 table regions to further train and evaluate layout segmentation models that focus on recognizing tabular data in historical domains.
• The dataset was created during the third phase of the OCR-D project [6]. During the project, transcription guidelines were developed together with the OCR-D community to ensure the interoperability and the reusability of ground truth datasets using three different transcription levels [7]. The transcriptions of our dataset rely on level 2 of the OCR-D Ground Truth Guidelines and can therefore be combined and reused with other datasets that follow the same transcription conventions.
• The dataset provides full texts for historical newspapers in very high quality. For this reason, it can serve as an ideal source for NLP workflows.

## 2 Background

The heightened interest in historical newspapers from the First (1914–18) and Second World War (1939–45), which offer daily news on economics, science, societal opinions and war events, underscores the importance of robust digitization. Despite the abundance of digitized newspapers, challenges persist in extracting high quality full texts, particularly for early 20th century glyphs like currency symbols and long (ſ), an omnipresent glyph in German books, periodicals and documents printed in Fraktur. Tabular data extraction from newspaper articles faces a similar challenge due to the scarcity of available datasets that can be used to train robust layout segmentation models. These challenges form the background for our dataset, which concentrates on the accurate transcription of historical newspapers and the representation of tabular data.

## 3 Data Description

**Source images:** The dataset [4] provides 101 files in PAGE XML format that capture layout segmentation and transcriptions for 101 source images. The source images are scans of newspaper pages of the historical newspaper “Deutscher Reichsanzeiger und Preußischer Staatsanzeiger” (German Imperial Gazette and Prussian Official Gazette), which was published from 1819 to 1945 under six different names and published as a digital edition in 2016 (shared under the Public Domain Mark 1.0 license) [1,8]. The dataset does not include the source images directly but rather enables the download of all images by using the bash script “download_images.sh”, located in the “data” folder of the Zenodo repository.

**Ground truth in PAGE XML format**: The layout segmentation and transcriptions matching the source images are provided as individual PAGE XML [9] files in the “data” folder. For each of the 101 source images, there is a PAGE XML file available in two different variants. These variants are:

**Variant 1: Ground truth in PAGE XML format (with table regions):** The folder “reichsanzeiger-1820–1939_with-TableRegion” provides all 101 PAGE XML files with annotated table regions.

**Variant 2: Ground truth in PAGE XML format (with table regions transformed to text regions)**: The folder “reichsanzeiger-1820–1939” provides all 101 PAGE XML files without table regions. All existing table regions were transformed to text regions using Transkribus’ built in “Transform tables to region” function. This function converts tables in such a way that each table cell is transformed to an individual text region. Hence the significant difference between the text region counts in variant 1 and 2.

**Documentation**: Further project documentation can be found at GitHub [10]. Metadata, containing project details, staff, transcription guidelines and sources can be found in the “METADATA.yml” of the Zenodo repository [4]. Overall statistics (e.g., glyph distributions for the whole dataset) can be found at GitHub as well [11].

## 4 Experimental Design, Materials and Methods

**Image selection**: Out of the 361,713 available scans at [1] we manually selected 96 double and 5 single newspaper pages for the years 1820–1939. The pages were selected for the following reasons: 1) A representative page layout with common layout components of historical newspapers, i.e., header, headings, text paragraphs, tables, and lists. 2) A wide range of different knowledge domains, e.g., politics, economics, culture, and official announcements. 3) A representative time period that covers both the changes in fonts and the changes in newspaper layout and contents.

**Fig. 1:**
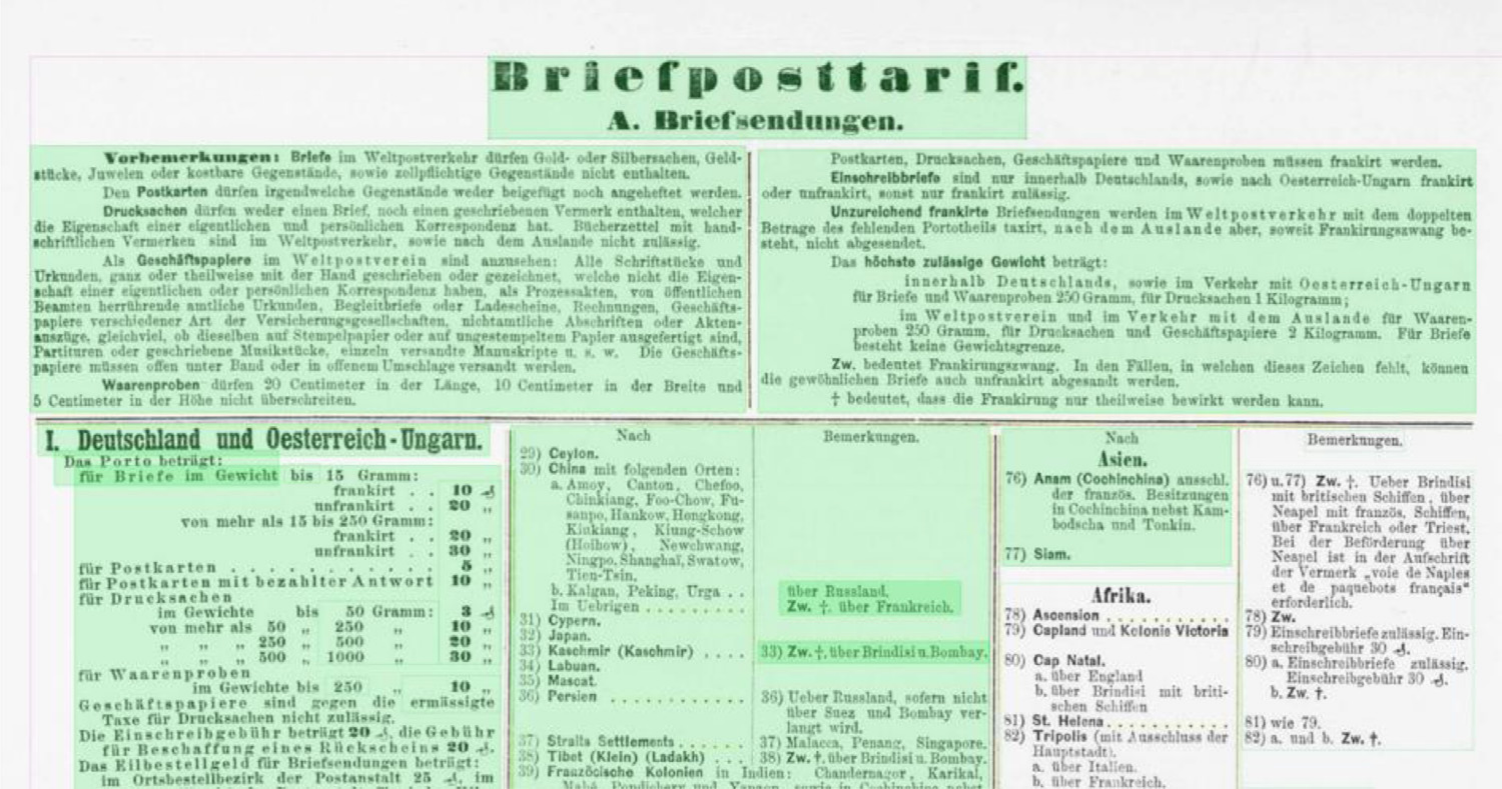
A newspaper page after applying Transkribus’ block detection. Some of the automatically created text regions are selected and highlighted in green. Especially the tabular data is recognized poorly compared to Fig. 2, as individual columns, rows and cells are frequently represented by single and/or overlapping text regions.

**Fig. 2:**
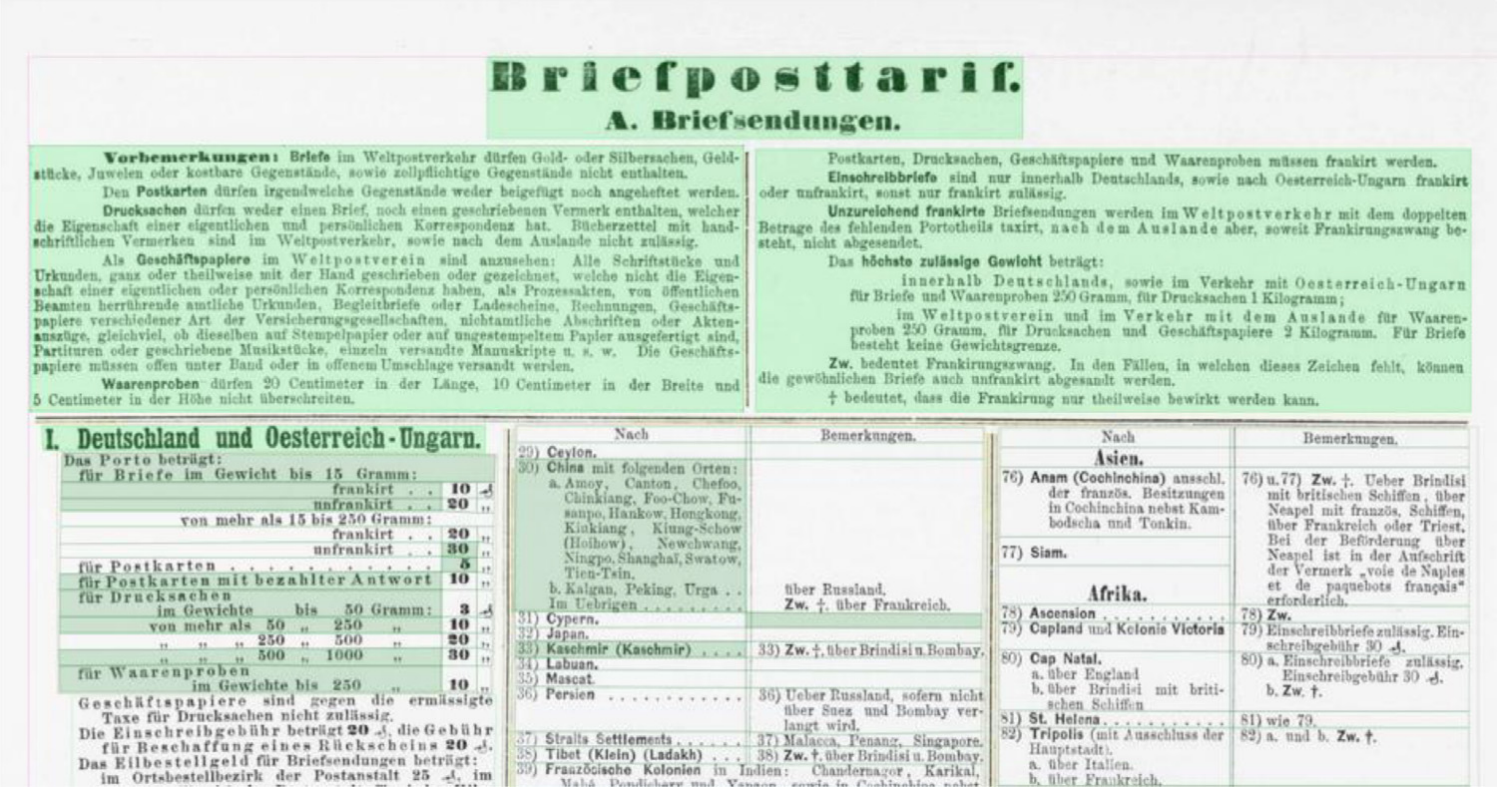
The same page as in Fig. 1 with manually created table regions. Some of the table cells were selected to highlight the correct representation of the given tabular data.

**Fig. 3:**
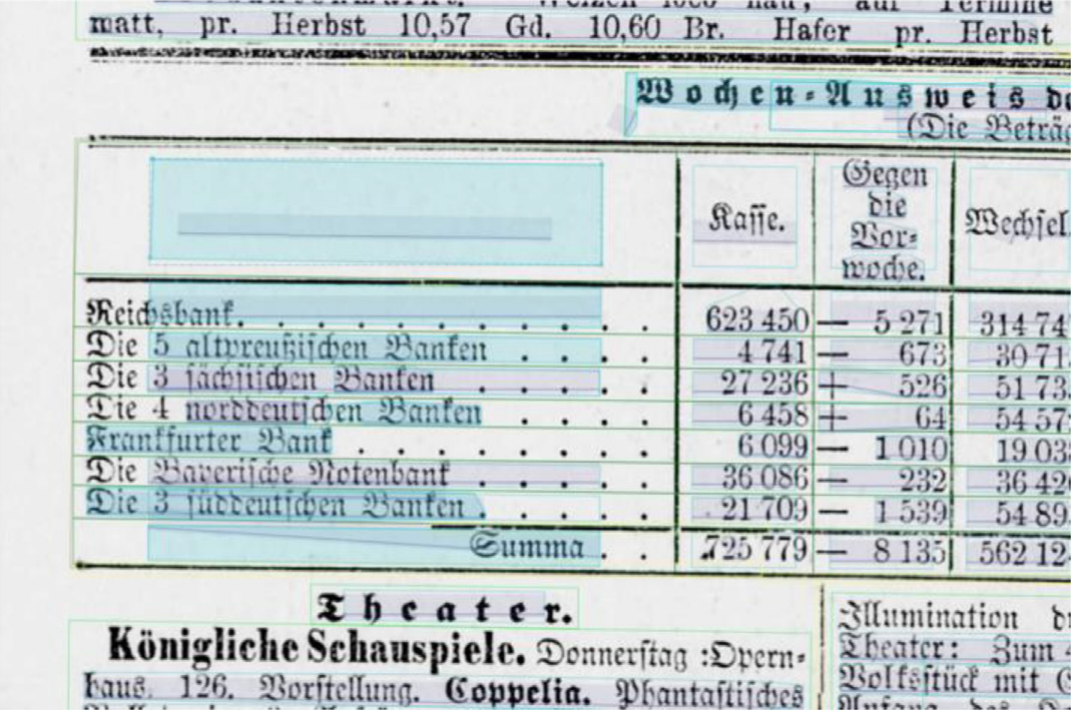
A newspaper page with incorrectly captured text line bounding boxes highlighted in blue.

**Fig. 4:**
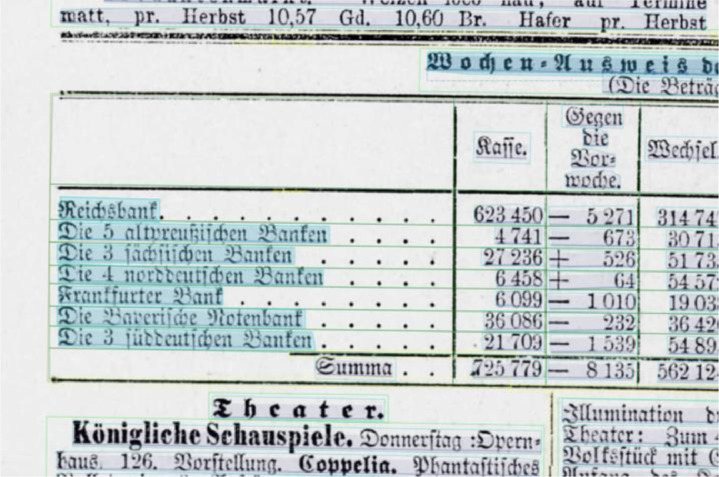
The same page as in Fig. 3 with manually corrected text line bounding boxes.

**Layout segmentation**: Due to layout complexities we applied a granular workflow, using Transkribus [2] and Tesseract [3] as software tools to create the layout segmentation for all 101 pages. 1) Using Transkribus’ built in block detection, which is part of the software's layout analysis tools, we automatically generated text regions for all pages. 2) These automatically generated text regions were corrected manually, following guidelines that were based on the OCR-D Ground Truth Guidelines [7] as well as an iteratively adapted and project specific ruleset [12] so that the layout components of the newspaper page (headlines, paragraphs, etc.) could be captured as accurately as possible. Tables in particular were inadequately captured by Transkribus’ block detection, which is why all table regions were created manually (see Fig. 1, Fig. 2). 3) We then annotated all regions using a set of 5 structure types: header, heading, paragraph, table, reference [13]. 4) Using Tesseract, we automatically generated bounding boxes and baselines for all existing text lines, which were 5) manually corrected again (see Fig. 3 and 4). 6) We corrected the reading order for all regions.

**Transcriptions**: 1) After the finished layout segmentation we extracted full texts using Tesseract with frak2021 model trained by Mannheim University Library [15]. 2) The automatically extracted full texts were manually corrected in two runs by a team of four transcribers using Transkribus. Transcriber 1 corrected the transcriptions generated by Tesseract, while transcriber 2 corrected the manual corrections made by transcriber 1. Throughout this process the OCR-D Ground Truth Guidelines level 2 were used as transcription guidelines, since level 2 reproduces “the technical printing conditions […]”, while the “interpretation of signs is oriented towards their use in the language and writing system” [7]. Therefore, our dataset does not normalize historical glyphs like long s (ſ) or rotunda r (ꝛ) or the double oblique hyphen (⸗), commonly used to hyphenate words in historical texts typeset in Fraktur, to their modern equivalents like round s, normal r or a standard hyphen (-). In three special cases, we deviated from the OCR-D guidelines in order to capture certain glyphs true to the original. These cases include double oblique hyphen (⸗), em dash (—) instead of en dash (–), and asterisk (*) used for both standard asterisk (*) and tear-drop asterisk (✻) [14]. 3) Finally, we used the finished transcriptions from transcriber 2 to finetune a PyLaia model in Transkribus. Ensuring that the model avoided overfitting to the training material, it was then utilized to identify discrepancies within the finished transcriptions.

**Post processing**: After finishing the layout segmentation and transcriptions we exported two variants of all 101 pages from Transkribus in PAGE XML format [9]. Variant 1 contains all manually created table regions as table regions (cf. Table 1). Variant 2 contains all table regions transformed to text regions for easier processing (cf. Table 2).

**Table 1:** Number of regions, lines, words and glyphs for variant 1: Ground truth in PAGE XML format (with table regions).

Text regions	Table regions	Text lines	Words	Glyphs
4491	412	119,430	490,679	2967,330

**Table 2:** Number of regions, lines, words and glyphs for variant 2: Ground truth in PAGE XML format (without table regions).

Text regions	Table regions	Text lines	Words	Glyphs
65,563	0	119,430	490,679	2967,330

## Limitations

The table regions annotated in the dataset largely contain economic data, as tabular data was of particular interest for this project. The first half of the 19th century is underrepresented in comparison to the second half of the century, as only microfiches of poor quality were available for this period. Although the dataset also includes layout regions typical for (historical) newspapers, such as adverts, these are underrepresented in comparison to text regions. Furthermore, the dataset is based on a single newspaper, whose temporal changes in content, layout and typeface are captured by the dataset, but are not representative of the newspaper landscape of the 19th and early 20th century.

The structural annotation of the layout regions is currently rather coarse, as the ground truth was created as part of the OCR-D project, which main goal is to “facilitate research access” to the “Union Catalogue of Books of the 16th–18th century (VD 16, VD 17, VD 18)” [16]. As the OCR-D project is primarily focused on printed books, the ground truth guidelines developed during the project reflect this focus accordingly and do not cover detailed descriptions of structural layout elements found in newspapers. However, as soon as the OCR-D ground truth guidelines are extended for printed documents like newspapers they can be applied to the dataset in order to enhance the current structural annotations.

## Ethics Statement

The authors have read the ethical requirements for publication in Data in Brief and hereby affirm that the work did not involve the use of human subjects, animal experiments and/or data collected from social media platforms. The authors did not need permission to use the primary data as it is published under Public Domain Mark 1.0 [17].

## CRediT authorship contribution statement

**Thomas Schmidt:** Project administration, Validation, Data curation, Supervision, Writing – original draft. **Jan Kamlah:** Conceptualization, Methodology, Software, Validation, Data curation, Supervision, Project administration, Writing – review & editing. **Stefan Weil:** Software, Project administration, Writing – review & editing.

## Data Availability

reichsanzeiger-gt (Original data) (Zenodo).

## References

[1] S. Weil, “Deutscher Reichsanzeiger und Preußischer Staatsanzeiger” (digital edition), Universitätsbibliothek Mannheim, 10.7801/REICHSANZEIGER.

[2] https://readcoop.eu/transkribus/, last Accessed 26 January 2024.

[3] https://github.com/tesseract-ocr/tesseract, last Accessed 26 January 2024.

[4] Schmidt T., Kamlah J., Weil S., Shigapov R. (2023). UB-Mannheim/reichsanzeiger-gt: 1.0.0. Zenodo.

[5] Hashmi K.A. (2021).

[6] https://ocr-d.de/en/about, last Accessed 26 January 2024.

[7] https://ocr-d.de/en/gt-guidelines/trans/level_2_2.html, last Accessed 26 January 2024.

[8] Kling C. (2016). Deutscher Reichsanzeiger und Preußischer Staatsanzeiger: Einleitung zur Veröffentlichung der Digitalausgabe. Report in MADOC.

[9] Pletschacher S., Antonacopoulos A. (2010). 2010 20th International Conference on Pattern Recognition.

[10] https://github.com/UB-Mannheim/reichsanzeiger-gt, last Accessed 26 January 2024.

[11] https://github.com/UB-Mannheim/reichsanzeiger-gt/wiki/Statistics, last Accessed 26 January 2024.

[12] J. Kamlah, T. Schmidt, “Transkriptionsregeln und Guidelines zur Layoutbearbeitung im DFG-Projekt ‘Workflow für werkspezifisches Training auf Basis generischer Modelle mit OCR-D sowie Ground-Truth-Aufwertung'”, 2023, 10.5281/zenodo.10203335.

[13] Cf. Kamlah and Schmidt, “Transkriptionsregeln”, pp. 32–33.

[14] A full description of all captured special glyphs can be found in the file “METADATA.YML” here: https://github.com/UB-Mannheim/reichsanzeiger-gt/blob/main/METADATA.yml, last Accessed 7 November 2023.

[15] S. Weil, “Tesseract OCR models for historic prints based on Latin script”, Vers. 1, 2021, 10.5281/zenodo.10125246.

[16] https://ocr-d.de/en/about.html, last Accessed 26 January 2024.

[17] https://digi.bib.uni-mannheim.de/service/, last Accessed 26 January 2024.

